# Radiofrequency ablation of stage IA non–small cell lung cancer in patients ineligible for surgery: results of a prospective multicenter phase II trial

**DOI:** 10.1186/s13019-018-0773-y

**Published:** 2018-08-24

**Authors:** J. Palussière, F. Chomy, M. Savina, F. Deschamps, J. Y. Gaubert, A. Renault, O. Bonnefoy, F. Laurent, C. Meunier, C. Bellera, S. Mathoulin-Pelissier, T. de Baere

**Affiliations:** 1 Department of Interventional Radiology, Institut Bergonié, 229 Cours de l’Argonne, 33000 Bordeaux, France; 2 Department of Medical Oncology, Institut Bergonié, 229 Cours de l’Argonne, 33000 Bordeaux, France; 3 Department of Clinical and Epidemiological Research, Institut Bergonié, 229 Cours de l’Argonne, 33000 Bordeaux, France; 4 Department of Interventional Imaging, Institut Gustave Roussy, 114 Rue Edouard Vaillant, 94800 Villejuif, Paris, France; 5 Department of Imaging, CHU Timone, 264 Rue Saint-Pierre, 13385 Marseille, France; 6 Department of Imaging, CHU Pau, 4 Boulevard Hauterive, 64000 Pau, France; 7 Department of Imaging, CHU Haut Lévêque, Avenue Magellan, 33600 Pessac, France; 8 Department of Imaging, CHU Rennes, 2 rue Henri Le Guilloux, 35033 Rennes, France

**Keywords:** Ablation, Non-small cell lung cancer, Radiofrequency ablation, Stereotactic body radiotherapy

## Abstract

**Background:**

A prospective multicenter phase II trial to evaluate the survival outcomes of percutaneous radiofrequency ablation (RFA) for patients with stage IA non-small cell lung cancer (NSCLC), ineligible for surgery.

**Methods:**

Patients with a biopsy-proven stage IA NSCLC, staging established by a positron emission tomography-computed tomography (PET-CT), were eligible. The primary objective was to evaluate the local control of RFA at 1-year. Secondary objectives were 1- and 3-year overall survival (OS), 3-year local control, lung function (prior to and 3 months after RFA) and quality of life (prior to and 1 month after RFA).

**Results:**

Of the 42 patients (mean age 71.7 y) that were enrolled at six French cancer centers, 32 were eligible and assessable. Twenty-seven patients did not recur at 1 year corresponding to a local control rate of 84.38% (95% CI, [67.21–95.72]). The local control rate at 3 years was 81.25% (95% CI, [54.35–95.95]). The OS rate was 91.67% (95% CI, [77.53–98.25]) at 1 year and 58.33% (95% CI, [40.76–74.49]) at 3 years. The forced expiratory volume was stable in most patients apart from two, in whom we observed a 10% decrease. There was no significant change in the global health status or in the quality of life following RFA.

**Conclusion:**

RFA is an efficient treatment for medically inoperable stage IA NSCLC patients. RFA is well tolerated, does not adversely affect pulmonary function and the 3-year OS rate is comparable to that of stereotactic body radiotherapy, in similar patients.

**Trial registration:**

ClinicalTrials.gov Identifier NCT01841060 registered in November 2008.

## Background

Lobectomy and lymph node resection remains the first-line treatment and the best option for stage I non-small-cell lung cancer (NSCLC) [1]. Despite the development of sub-lobular resection [2] to limit the functional damage of lobectomy, approximately 20% of patients remain ineligible for surgery (mostly due to comorbidities). The 5-year overall survival (OS) without treatment ranges from 6 to 14% for these patients [3]. Until recently, these ‘non-surgical’ patients were treated with conventional radiotherapy [4]. However, current treatments such as stereotactic body radiotherapy (SBRT) [5–8] and thermal ablation [9–15] are increasingly offered as alternative therapies for non-surgical candidates. Radiofrequency ablation (RFA) has been the most commonly used and evaluated image-guided thermal ablation technique. RFA was shown to be feasible and safe in highly selected patients [9–14]. One of the advantages of RFA is that it is a stand-alone therapy which can be repeated in case of local failure. RFA has the ability to eradicate a tumor with heat while causing minimal damage to the surrounding normal lung tissue. The objectives of this multicenter study were to prospectively analyze the efficacy and the tolerance of RFA in stage I NSCLC for non-surgical patients.

## Methods

The study was approved by the ethics committee and was funded by the Programme Hospitalier de Recherche Clinique (PHRC, grant number A00812–53) and was conducted in six French institutions. This study followed Consolidated Standards of Reporting Trials guidelines.

### Objectives

The main objective was to evaluate the local control of RFA at 1 year. Secondary objectives were to estimate the overall survival (OS) at one and 3 years, local control at 3 years, lung function and quality of life (QoL) after RFA.

### Patients

#### Inclusion and exclusion criteria

Patient, clinical and follow-up data were prospectively registered. Prior to participation, all patients provided written informed consent. Eligible patients had biopsy-proven stage IA NSCLC with a maximum tumor diameter ≤ 3 cm; staging was established by a positron emission tomography-computed tomography (PET-CT) and contrast-enhanced CT. Fluorine-18 fluorodeoxyglucose PET-CT was performed no more than 8 weeks prior to inclusion. RFA was considered technically feasible following a discussion by the local thoracic tumor board. Tumor location < 1 cm from the hilum was the main technical contraindication for RFA. Patients with a tumoral standardized uptake value ≤2.5 on PET-CT were excluded. Patients were required to have an Eastern Cooperative Oncology Group (ECOG) performance status of 0 to 2. Lung insufficiency with forced expiration volume in the first second of expiration (FEV1) < 1 l was not an absolute contraindication.

#### RFA treatment

CT guidance was used to treat tumors under general anesthesia. Thoracic epidural anesthesia was administered in case of contraindication to general anesthesia mostly due to poor respiratory function. All patients were treated with the same multitine electrodes (LeVeen; Boston Scientific, Nattick. MA) measuring 3, 3.5, or 4 cm in diameter and at least 10 mm larger than the diameter of the target tumor. Multiple overlapping ablations were performed, when needed, in different parts of the tumor in order to cover the entire volume.

#### Imaging follow-up and complications

Patients were followed-up with a thoracic contrast enhanced CT imaging at 1, 3, 6, 9 and 12 months and a whole body contrast enhanced CT every 6 months. A PET-CT was performed at 3 months and 1 year. Whilst any decrease or stable size in CT images was considered as complete treatment, local control was deemed incomplete in case of > 20% increase in the size of the ablation zone in the largest diameter between two follow-up CTs, or appearance of any irregular, nodular foci at the margin of the ablation zone. On PET-CT, if the ablation zone uptake was greater than the mediastinal background, it was considered as a local recurrence [16]. All images have been remotely reviewed by an independent re-interpretation committee.

In case of a local recurrence, the patient file was reviewed by the tumor board to determine the best option. After 1 year, a PET-CT was proposed to evaluate local control or systemic progression. Complications observed during follow-up, either clinically relevant or observed in imaging, were noted for each patient. Adverse events, based on CTCAE 3.0 [17], were noted and attributed to the study treatment if the physician believed so. Any patient death within 30 days after RFA was considered a grade 5 adverse event. Grade 3 or 4 adverse events were defined as major complications and grade 1 or 2 as minor complications.

#### Evaluation criteria/ analysis

The main objective was to evaluate local tumor control rate at 1 year, local control defined as the absence of progression of the ablated site. In case of discordance between CT and PET-CT at 1 year, a biopsy was performed. In order to obtain a homogeneous interpretation and to validate the results, one-year imaging data were reviewed at the end of the study by an independent committee composed of a radiologist, a nuclear physicist and a thoracic oncologist. The local control rate was calculated as the ratio of the number of patients alive without local progression at 1 year. A sensitivity study was performed by including eligible patients who had received the RFA treatment and lost to follow-up or deceased before their 1-year evaluation. Based on their last follow-up evaluation before 1 year, patients were deemed locally progressive or not, and the corresponding local control rate was calculated. Confidence intervals of the local control rates were estimated by the Fisher approximation method based on the sample size.

Survival endpoints were defined according to the DATECAN guidelines [18]. OS was defined as the time from RFA treatment to death, whatever the cause. If the patient was still alive at the end of study or lost to follow-up, the patient was censored at the date of last news. PFS was defined as the time from RFA treatment to disease progression or death, whatever the cause. If the patient was still alive with no progression at the end of study or lost to follow-up before observation of a progression, the patient was censored at the date of last news.

Survival endpoints were evaluated using the Kaplan-Meier method and survival rates are reported with their standard error (SE).

Lung function tests including FEV1, forced vital capacity (FVC), total lung capacity (TLC), were performed prior to and 3 months following RFA. A 10% decrease in FEV1 and FVC, and a 20% TLC decrease were considered significant. QoL was measured prior to and 1 month following RFA with the QLQ C 30 questionnaire, developed by the EORTC [19]. The questionnaire consists of 30 items that allow estimating 5 functional and 5 symptomatic scores as well as a global health status score (between 0 and 100). A score of 100 corresponds to a perfect QoL, whereas a score of 0 reflects a very poor QoL. On the contrary, on the symptomatic scale, a score of 100 corresponds to a very poor QoL and a score of 0 reflects a perfect QoL. A 10 point increase or decrease between the pre- and the post-RFA evaluations were considered significant.

#### Statistical analysis

The number of patients to include was assessed following a binomial law, considering a 5% type-one error and 85% statistical power. In order to observe a 1-year local control rate statistically superior to 65%, with the hypothesis that it will reach 85% in the experimental RFS arm, 33 assessable patients were necessary. Taking into account eventual losses (patients lost to follow-up or deceased before 1 year), at least 40 patients needed to be included.We reported confidence intervals for a 95% two-sided confidence-level (95% CI), estimated using the binomial approach. All statistical analyses were performed using SAS software v9.3 and figures were generated using STATA software and Microsoft Excel software.

## Results

**Fig. 1:**
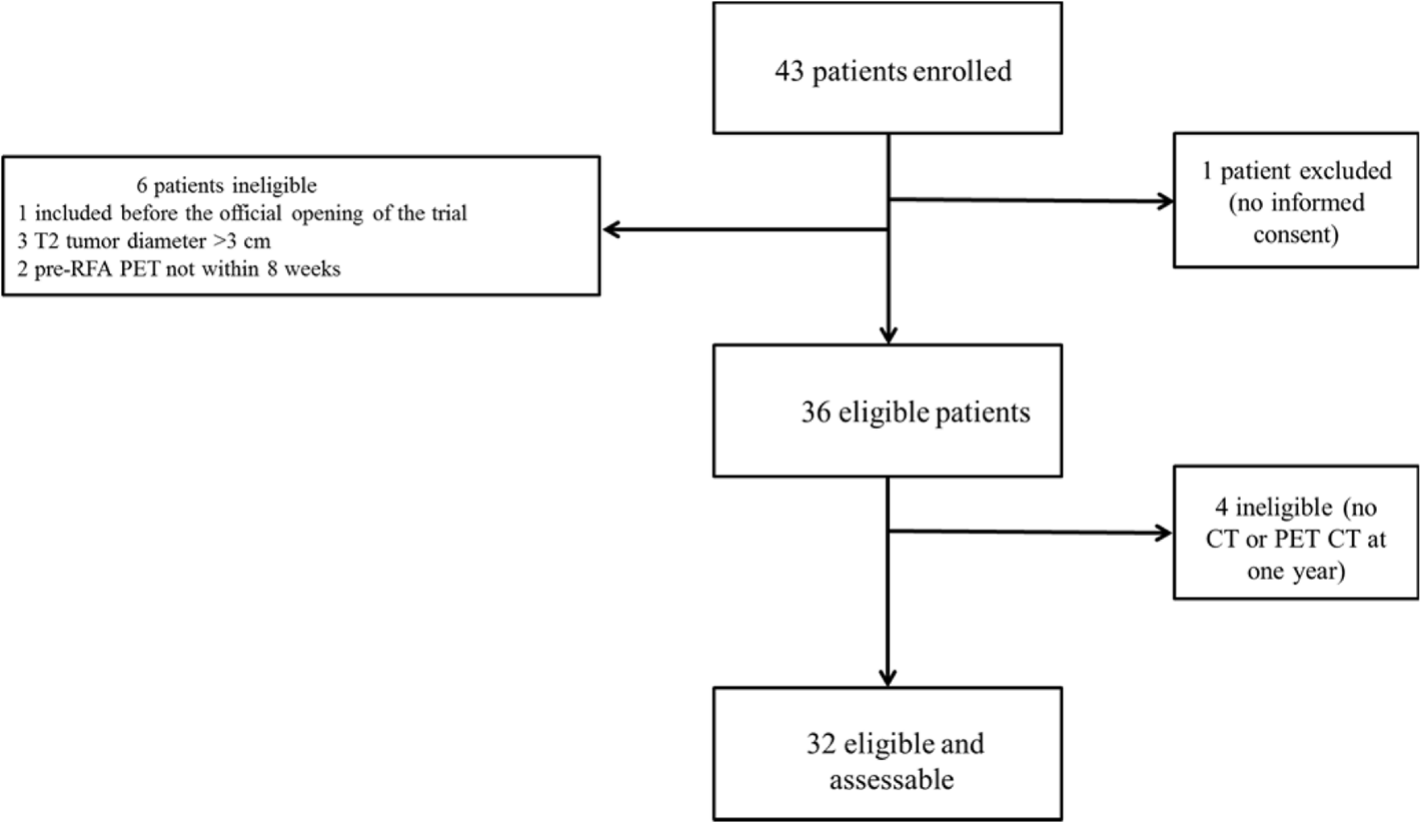
Flowchart of patient inclusion

Patient characteristics are given in Table 1. A total of 42 patients were included in the study out of which 36 patients were eligible and 32 patients were eligible and assessable for the main criteria (Fig. 1). Majority of patients were ECOG1 and the tumor characteristics (pathology, dimensions etc.) are presented Table 1. Histological analysis revealed an adenocarcinoma in 60% of the patients. T1a and T1b tumors were almost equally distributed. PET-CT at inclusion revealed that SUVmax ranged from 2.7 to 22.2 (mean = 6.9; median = 6.7) for all patients and from 2.7 to 14.5 (mean = 6.3; median = 5.4) for assessable patients. RFA treatments were performed under general anesthesia, except for 4 interventions where thoracic epidural anesthesia was administered. A 4 cm electrode was used in 60% of patients, even though half of the tumors were T1a. Moreover, overlapping ablations were frequent and were performed in more than half of the tumors. A 48 h hospitalization was systematically planned for every patient as part of the protocol for this old and fragile population with multiple comorbidities. Hospitalization duration ranged from 2 to 19 days (mea*n* = 4 days; median = 3 days). An X-ray for pneumothorax was performed during ablation in 26 treatments (83.87%), with the need for a chest tube in 17. Chest tubes were always removed within 2 days. Other CT findings were pleural effusion (n = 4; 1 mild and 3 minimal), alveolar hemorrhage (*n* = 2; subsegmental), hemothorax (*n* = 1); however, no clinical symptoms were noted in relation to these findings. Two major adverse events were recorded in 2 patients (4.7% of the overall population) and 3 delayed minor adverse events were noted (Table 2).

**Table 1:** Patient characteristics

	Population *n* = 42	Eligible population *n* = 36	Eligible and assessable population *n* = 32
Men	29	23	21
Women	13	13	11
Mean Age (years)	71.7	72.8	72.7
ECOG Status			
0	11	10	9
1	23	20	17
2	1	0	0
Non-documented	7	6	6
Smoking			
No	5	5	5
Yes	23	18	16
Non-documented	14	13	11
Histological analysis			
Squamous cell carcinoma	10	9	8
Adenocarcinoma	26	23	21
Large cell carcinoma	4	3	3
Undifferentiated non-small cell	1	1	0
Neuroendocrine	1	0	0
Tumor size (mm)			
Max.	37	29	29
Min.	13	13	13
Average	21.1	20.4	20.7
STD	6.34	5.04	5.0
Tumor stage			
T1a	24 (57%)	21 (58%)	18 (56%)
T1b	15 (36%)	15 (42%)	14 (44%)
T2	3 (7%)	0 (0%)	0 (0%)

**Table 2:** Adverse Events

Description	Grade CTCAE	Nb	Evolution	Imputability
Pulmonary embolism	4	1	Healing without sequelae	Non-attributable
Acute lung insufficiency	5	1	Death	Attributable
Dyspnea	2	1	Healing without sequelae	Non-attributable
Dyspnea	2	1	Healing with sequelae	Attributable
Rib lysis and fracture	1	1^a^	Healing	Attributable

^a^2nd right rib was concerned and the fracture occurred 6 months after RFA, the ablation zone was in contact with the chest wall

### Local control (Table 3)

Local control was observed in 27 of the 32 eligible patients assessable for efficacy corresponding to a local control rate of 84.38% (95% CI, [67.21–95.72]). Among the 5 diagnosed with local progression, 1 required biopsy to prove the local failure due to a discrepancy in the interpretations of PET-CT and CT. A second RFA was performed in two of the 5 local progression cases. The local control rate at 3 years was 81.25% (95% CI, [54.35–95.95]). A sensitivity study was performed including all eligible patients and the local control rate was 86.11% [95% CI, [70.50–95.33]) at 1 year and 77.78% [95% CI, [60.85–89.88]) at 3 years.

**Table 3:** Different recurrences

Type of recurrence	Number
Local failure (ablation area)	5
Lymph node (hilar)	2
Lymph node N2	2
Lymph node N3	1
Metastatic disease	1
Lymph node + metastatic disease	1
Recurrence within another lobe + Lymph node	1

### Secondary objectives

#### OS and progression free survival (PFS)

**Fig. 2:**
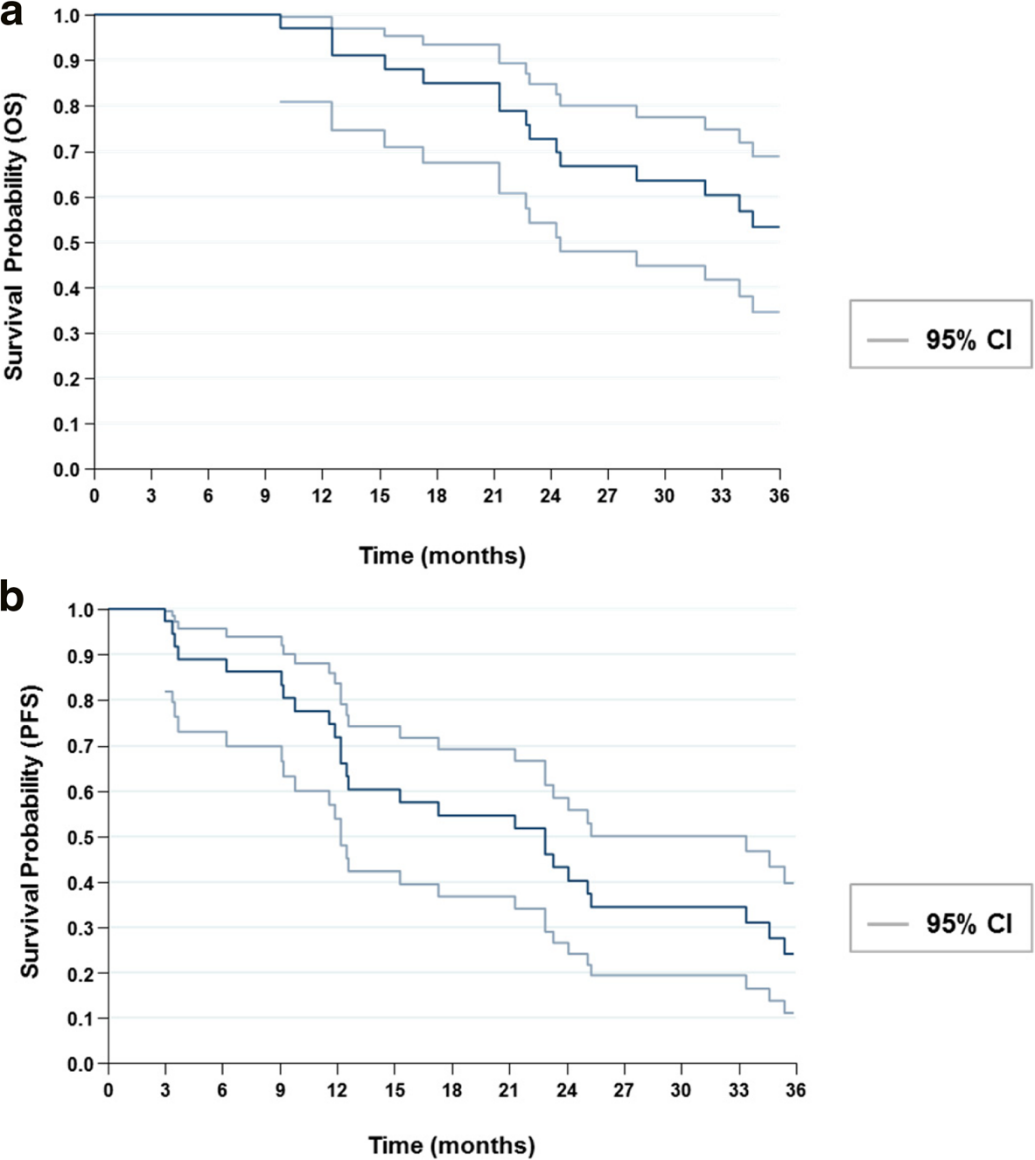
**a** Overall survival and (**b**) Progression-free survival curves

The OS was 91.67% (95% CI, [77.53–98.25]) at 1 year and 58.33% (95% CI, [40.76–74.49]) at 3 years (Fig. 2). PFS was 71.76% (95% CI, [42.25–88.00]) at 1 year and 25% (95% CI, [12.12–42.20]) at 3 years. Fifteen patients died within the 3 years follow-up (7 from cancer progression and 8 from other causes) and two patients died before 1 year (1 from cancer progression).

#### Lung function

Of the 36 eligible patients, FEV1 was available for 21 patients prior to treatment and for 18 patients at 3 months. Prior to treatment, FEV1 ranged from 0.6 to 35.7 (mean = 3.2; median = 1.6) whereas at 3 months FEV1 ranged from 0.6 to 3.2 (mean = 1.6; median = 1.6).

If we only focus on the 14 patients assessed both prior to and after treatment, pre-treatment FEV1 ranged from 0.9 to 35.7 (mean = 4.2; median = 1.7) and three-month FEV1 ranged from 0.9 to 3.2 (mean = 1.7; median = 1.6). Two patients had 10% decrease and two patients had a 10% increase in FEV1.

Regarding FVC, pre-treatment and three-month assessment was available for 20 and 17 patients, respectively. Prior to treatment, FVC ranged from 1.5 to 4.3 (mean = 2.6; median = 2.3). Three months after RFS, FVC ranged from 1.6 to 4.9 (mean = 2.7; median = 2.3). If we only consider the 13 patients assessed both prior to and 3 months after treatment, pre-treatment FVC ranged from 1.7 to 4.3 (mean = 2.6; median = 2.3) and post-treatment FVC ranged from 1.6 to 4.9 (mean = 2.8; median = 2.3). Lung function test was available for 14 of the 36 eligible patients, with a 10% decrease in FEV1 in two patients and a 10% increase in two patients. A 20% decrease was observed in one patient and a 20% increase in two patients. TLC was available for nine patients and a 20% decrease was observed in one patient.

#### Quality of life

**Fig. 3:**
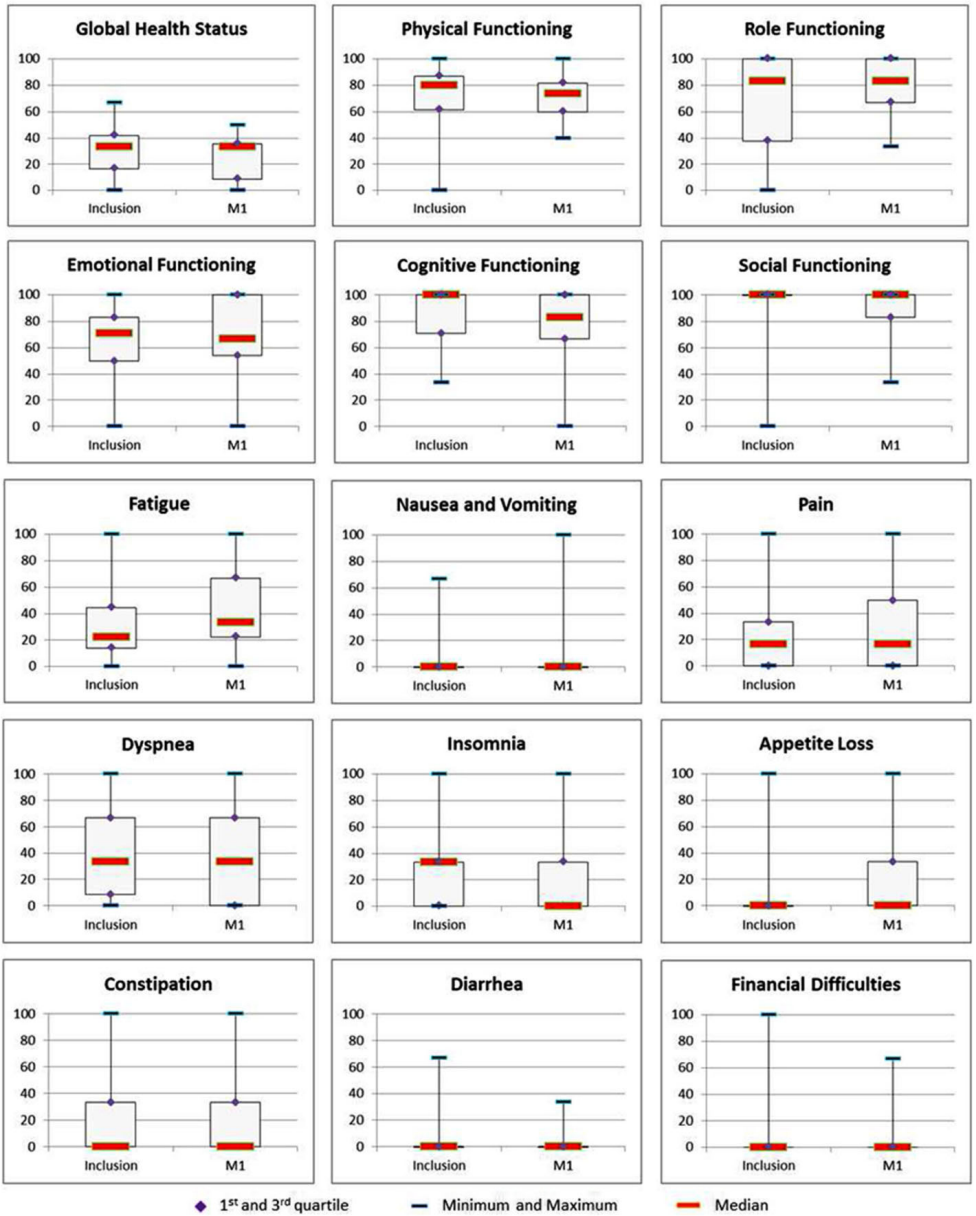
QLQ C30 boxplots depict changes in global health status, functional scales and symptomatic scales

QLQ C 30 questionnaires were available for 34 of the 36 eligible patients and the results are presented in Fig. 3. Between inclusion and 1 month, a significant deterioration of cognitive functions (decrease of 17.67 points) and fatigue (increase of 11.11 points) were observed, whereas insomnia significantly decreased (down by 33.33 points). It is worth noting that no significant modification of the median global health status, physical functioning, pain and dyspnea occurred.

## Discussion

**Fig. 4:**
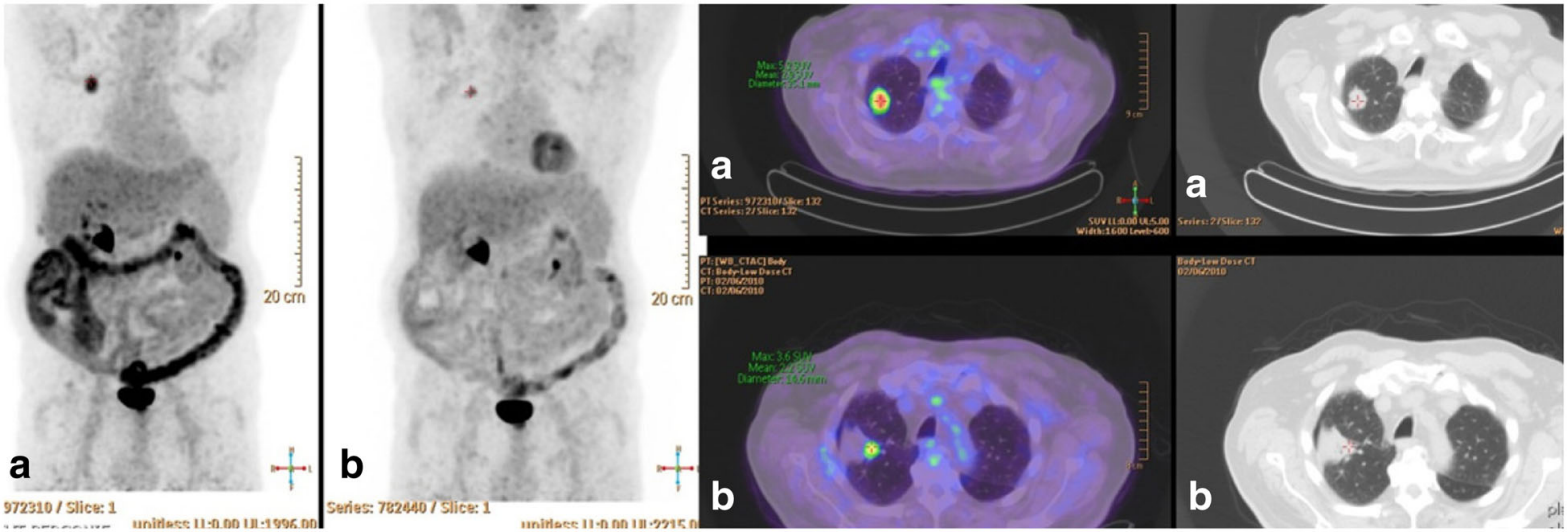
PET-CT before (**a**) and after RFA (**b**): an incomplete treatment can be seen on the edge of the ablation zone

This multicenter study is the second prospective study on RFA and non-surgical patients with stage I NSCLC. Dupuy et al. have published [20] the results of American College of Surgeons oncology group Z4033 trial, with a local recurrence-free rate of 68.9 and 59.8% at 1 and 2 years, respectively. In our study, the local control rate was 84.38% (95% CI, [67.21–95.72]) at 1 year and 81.25% (95% CI, [54.35–95.95]) at 3 years. In our study, local recurrence refers to a recurrence in the RFA zone (Fig. 4) whereas in the Z4033 trial it refers not only to a recurrence in the RFA site but also in the primary tumor lobe and the hilar lymph node. Our results are in accordance with our previously published retrospective study, with local control rates of 88.5 and 78.9% at 1 and 3 years, respectively [21].

In this study, expandable multitined electrodes were used with an electrode array diameter of the at least 10 mm larger than the tumor, such oversizing the ablation volume was previously reported to increase the success rate of the treatment [22]. Moreover, a pathological study following RFA on NSCLC demonstrated the benefit of oversizing the ablation zone by encompassing the tumor; < 5 mm margins were observed in patients with incomplete ablation whilst a mean margin of 8 mm was noted in complete ablations [23]. This emphasizes the need for an extensive RFA to take into consideration the microscopic extension of NSLCC. Beland et al. [10] recommended an ablation zone “*that includes the primary tumor plus at least an additional 8–10 mm of ablation beyond the visible tumor margin in all directions*”. In our series, all the patients apart from three were treated with a 3.5 or a 4 cm electrode for a mean tumor size of 20.7 mm, probably explaining the good high rate of local control we report herein. Other ablation techniques such as microwaves may also improve the local control rate by providing a larger ablation volume compared to RFA, as reported in an animal study [24]. Cryotherapy [25], offers the possibility of using multiple probes to obtain a larger ablation volume.

Ever since the use of thermal ablation on lung tumors, different studies [10, 23, 26] have shown that repeated imaging is important to follow the variations in size of the ablation zone, to identify the different imaging patterns of lung tumors after RFA and finally, to distinguish total ablation from local tumor progression. The strength of this study is the strict prospective continuous imaging follow-up during 3 years, for all patients. Recurrence was determined using CT and PET-CT, as well as the possible use of biopsy. In one case, there was discordance between CT and PET-CT at 1 year, a local recurrence was confirmed by a biopsy. A new RFA was proposed for 2 out of 5 local failures. Moreover, with the possibility of re-treatment, loco-regional progression did not seem to negatively impact the OS [27]. The 1- and 3-year OS rates were of 91.67 and 58.33%, respectively which are similar to the American prospective trial results [20] with an OS rate of 86.3 and 69.8% (at 1 and 2 years, respectively). Furthermore, our patient population was old, frail and contraindicated for surgery. We note that RFA results are difficult to compare with those of sub-lobular resection and Stereotactic Body Radiation Therapy (SBRT). Patients for thermal ablation are often the frailest and the oldest [28], in this series all patients were medically inoperable and at 3 years, half of the patients died of causes other than cancer. Nevertheless, SBRT resulted in similar survival rates (86 and 45% at 1 and 3 years, respectively) in an elderly population [6]. The local control rate at 3 years was 89% which is comparable to ours. However, in this series at 12 months of follow-up, imaging (CT scans) was available for 88% of patients, and PET-CT was obtained only when there was suspicion of disease recurrence. Moreover, histological confirmation of malignancy was available only for 39% of patients vs 100% in our series. The absence of pathological confirmation of cancer in a significant number of patients may raise the concern of whether those lung lesions may be benign. This is a weakness of numerous SBRT studies. Comparison with surgery is difficult as patients treated with ablation are not surgical candidates. Nevertheless, using propensity scores to match patient subgroups, Kwan et al. recently demonstrated comparable overall and lung-cancer specific survival between RFA and sub-lobular resection in patients ≥65 years old with stage IA or IB NSCLC [29]. The lack of pathological mediastinal lymph node information, which may impair patient survival compared to surgery, remains a limitation of RFA and SBRT for early stage NSCLC.

RFA seems to have been well tolerated. No delayed pneumothorax occurred and all pneumothoraces cleared rapidly. In a large series of 1000 RFA sessions [30], pneumothorax requiring pleural sclerosis was rare (1.6%). Besides pneumothorax, we observed two major adverse events. An ECOG 2 patient with a FEV of 800 mL died a few weeks later from respiratory decompensation that has been attributed to RFA. Exacerbation of interstitial pneumonia has been mentioned as a major cause of death [30] and has also been reported after lung surgery and radiotherapy. Consequently, the indication of a thermal ablation must be carefully weighted in patients with lung insufficiency, even though it is difficult to propose a clear lower threshold for respiratory function for lung RFA [23]. The stable lung function and the positive impact on QoL in our series also highlight the good tolerance of the RFA. QLQ C 30 tests propose large scale measurements and are not focused on lung disorders. A majority of patients considered that RFA did not modify either their life status perception or their main vital function, including lung function. A significant deterioration of cognitive function and fatigue was observed.

## Conclusion

This prospective study confirms that RFA is an effective, safe and well-tolerated option in the treatment of patients presenting stage IA NSCLC who are ineligible for surgery. The 3-year local control rate is encouraging and similar to SBRT results. The survival is comparable to another recent prospective study confirming the place of thermal ablation in this fragile non-surgical population. A prospective trial comparing RFA to other techniques such SBRT is required.

## Abbreviations

ECOG: eastern cooperative oncology group
FEV1: first second of expiration
FVC: forced vital capacity
NSCLC: non-small cell lung cancer
OS: overall survival
PET-CT: positron emission tomography-computed tomography
PFS: progression-free survival
QoL: quality of life
RFA: radiofrequency ablation
SBRT: stereotactic body radiotherapy
TLC: total lung capacity
